# UDCA Inhibits Hypoxic Hepatocellular Carcinoma Cell–Induced Angiogenesis Through Suppressing HIF-1α/VEGF/IL-8 Intercellular Signaling

**DOI:** 10.3389/fphar.2021.755394

**Published:** 2021-12-15

**Authors:** Wanfu Lin, Shu Li, Yongbin Meng, Guokai Huang, Shufang Liang, Juan Du, Qun Liu, Binbin Cheng

**Affiliations:** ^1^ Oncology Department of Traditional Chinese Medicine, Changhai Hospital, Naval Medical University (Second Military Medical University), Shanghai, China; ^2^ Faculty of Traditional Chinese Medicine, Naval Medical University (Second Military Medical University), Shanghai, China; ^3^ Department of Gastroenterology, Baoshan Hospital of Integrated Traditional Chinese and Western Medicine, Shanghai University of Traditional Chinese Medicine, Shanghai, China

**Keywords:** angiogenesis, hepatocellular carcinoma, hypoxia, HIF-1α, IL-8

## Abstract

**Background:** A hypoxic microenvironment may induce angiogenesis and promote the development of hepatocellular carcinoma (HCC). The aim of this study was to evaluate whether ursodeoxycholic acid (UDCA) may inhibit hypoxic HCC cell–induced angiogenesis and the possible mechanisms.

**Methods:** Tube formation and matrigel plug angiogenesis assays were used to evaluate angiogenesis *in vitro* and *in vivo,* respectively. Real-time PCR, enzyme-linked immunosorbent assay, and Western blot were used to evaluate the mRNA and protein expressions of hypoxia-inducible factor-1α (HIF-1α), vascular endothelial growth factor (VEGF), and IL-8, respectively. Dual-luciferase reporter assay was applied to assess the reporter gene expression of hypoxia-response element (HRE).

**Results:** UDCA antagonized hypoxic Huh 7 cell-induced tube formation of EA.hy 926 cells. In HCC cells, UDCA inhibited hypoxia-induced upregulation of VEGF and IL-8 both in mRNA and protein levels. UDCA also inhibited IL-8–induced angiogenesis *in vitro* and *in vivo* through suppressing IL-8–induced phosphorylation of ERK. The levels of HIF-1α mRNA and protein and HRE-driven luciferase activity in HCC cells were upregulated by hypoxia and were all inhibited by UDCA. The proteasome inhibitor MG132 antagonized the effect of UDCA on HIF-1α degradation. In hypoxic condition, the phosphorylation of ERK and AKT was obviously increased in HCC cells, which was suppressed by UDCA. Transfection of the HIF-1α overexpression plasmid reversed the effects of UDCA on hypoxic HCC cell–induced angiogenesis, HRE activity, and expressions of IL-8 and VEGF.

**Conclusions:** Our results demonstrated that UDCA could inhibit hypoxic HCC cell–induced angiogenesis through suppressing HIF-1α/VEGF/IL-8–mediated intercellular signaling between HCC cells and endothelial cells.

## Introduction

Hepatocellular carcinoma (HCC) is one of the most lethal malignancies with poor prognosis. Although more and more treatment options appeared with the development of experimental and clinical studies in the past decade, it is still highly refractory even after radical resection or ablation, and about 70% of patients experience tumor recurrence within 5 years (Nakagawa et al., 2016; Raoul and Edeline, 2020; Sung et al., 2021). Once progressed into the advanced stage, the overall survival (OS) is very limited, and the estimated 5-year OS is about 10% (Cabibbo et al., 2010; Rimassa et al., 2018). Thus, there is an urgent requirement to develop new therapeutic agents to improve the prognosis of the patients with HCC.

A hypoxic microenvironment is one of the most important characteristics of solid tumors, including HCC (Zhao et al., 2020). Hypoxia usually occurs when the tumor grows fast and the newly generated vessels are insufficient to provide enough oxygen for the tumor growth (Su et al., 2019). In HCC, transcatheter arterial chemoembolization (TACE), a usually used treatment method which aims to block the tumor blood supply and achieve the goal of tumor necrosis, also leads to local hypoxia (Lin et al., 2021). In addition, the anti-angiogenic therapy may also aggravate tumor hypoxia because of excessive inhibition of neovascularization (Wang et al., 2021). Hypoxia not only promotes the migration and invasion of tumor cells but also causes the upregulation of angiogenic factors, such as vascular endothelial growth factor (VEGF), which could stimulate the formation of tumor vessels (Chen et al., 2019). Therefore, the agents targeting hypoxia may be good candidates for HCC treatment.

Ursodeoxycholic acid (UDCA), a licensed first-line therapeutic agent for primary biliary cholangitis (Hirschfield et al., 2018), may decrease cholesterol saturation in the bile, solubilize cholesterol gallstones, and improve liver functions in case of cholestatic diseases (Tonin and Arends, 2018). Recent studies indicated that it may also prevent gallstone formation after bariatric surgery (Magouliotis et al., 2017) and is used for cystic fibrosis–related liver diseases (Cheng et al., 2017). Moreover, UDCA was reported to be able to inhibit various cancers such as colon cancer and HCC through anti-proliferation (Khare et al., 2003; Chung et al., 2011). It may also induce HCC cell apoptosis by regulating the ratio of Bax/Bcl-2, the expression of Smac and Livin, and the expression and activity of caspase-3 (Zhu et al., 2014). In a phase III, double-blind placebo-controlled trial, UDCA produced a statistically significant 39% reduction in recurrence of colorectal adenomas with high-grade dysplasia (Alberts et al., 2005). However, whether UDCA may prevent the neovascularization of HCC in a hypoxic microenvironment remains to be further investigated. Therefore, in the present study, we aimed to study whether UDCA may inhibit the angiogenesis of HCC under hypoxic conditions and the possible underlying mechanisms, which may provide some experimental basis for the future use of UDCA for the prevention and treatment of HCC.

## Materials and Methods

### Cell Culture and Reagents

The human HCC cell line Huh 7, HCC-LM3, and the human endothelial cell line EA.hy 926 were purchased from the Cell Bank of Chinese Academy of Sciences, Shanghai, China. According to the protocols, cells were cultured in Dulbecco’s modified Eagle medium (DMEM) supplemented with 10% fetal bovine serum (FBS) at 37°C in a humidified incubator with 5% CO_2_.

### MTT Assay for Cell Proliferation

Huh 7 cells or EA.hy 926 cells were seeded in 96-well plates with a density of 5 × 10^3^ cells/well. After 24 h, the cells were treated with different concentrations of UDCA (25, 50, 100, 200, and 400 μM) and cultured for another 24 h or 48 h. For the co-culture assay, EA.hy 926 cells were cultivated on the 24-well plate and Huh 7 cells were exposed indirectly to the cells by cultivating on the polycarbonate membrane of the 24-well transwell and treated with 50 μM UDCA for 24 h. Then, 10 µL of the MTT solution (5 mg/ml) was added into each well and incubated for an additional 4 h at 37°C. The medium was carefully removed, and 150 µL of DMSO was added and incubated overnight. Absorbance was measured at 570 nm using an EL × 800 microplate reader (BioTek, Norcross, GA, United States).

### Tube Formation Assay

Matrigel (BD bioscience) was thawed on ice at 4°C overnight before use and then was added into the pre-chilled 96-well or 24-well plates with 50 μL/well or 200 μL/well and incubated at 37°C for at least 30 min, allowing a gel to form. Then, 100 μL or 400 μL of EA.hy 926 cells (2 × 10^5^ cells/mL) were seeded in the plate alone or co-cultured indirectly with Huh 7 or HCC-LM3 cells (2 × 10^4^ cells in 100 μL culture medium) treated with different concentrations of UDCA (25 and 50 μM). A hypoxia incubator (1% O_2_, 94% N_2_, and 5% CO_2_) or CoCl_2_ (50 μM) was applied to induce hypoxia-like conditions. After 16-h incubation, capillary-like structures were captured through a microscope system (Leica, Germany). The number of junctions, total segment lengths, and mean mesh size were calculated with the open source software ImageJ (version 1.51) to quantify the tube formation.

### Real-Time RT-PCR

Total RNA was isolated with the TRIzol reagent (Invitrogen, Carlsbad, CA, United States), as described previously (Zhang et al., 2014), and cDNA was generated using the Prime Script RT-PCR kit (Takara Bio Dalian, China). Quantitative RT-PCR was performed in a CFX96 real-time system (Bio-Rad, CA, United States) using specific sense and antisense primers in 20 μL reaction volumes containing 10 μL of 2 × SYBR Green PCR master mix (Toyobo, Osaka, Japan), 6 µL of RNase free ddH_2_O, 2 µL of cDNA, 1 µL of forward primer, and 1 µL of reverse primer. Primers used for PCR were as follows: β-actin, forward: 5′-AGC GGG AAA TCG TGC GTG -3′, reverse: 5′-CAG GGT ACA TGG TGG TGC C-3′; HIF-1α, forward: 5′-TTC CCG ACT AGG CCC ATT C-3′, reverse: 5′-CAG GTA TTC AAG GTC CCA TTT CA-3′; VEGF, forward: 5′-GCC TCG GGC TTG TCA CAT TTT-3′, reverse: 5′-CCC TGA TGA GAT CGA GTA CAT CT-3'; IL-8, forward: 5′-TCT TGG CAG CCT TCC TGA TT-3′, and reverse: 5′-TGG TCC ACT CTC AAT CAC TCT CAG T-3′. Reaction parameters were as follows: 95°C for 3 min for denaturation and 40 cycles of 95°C for 10 s, 60°C for 20 s, and 72°C for 25 s. The relative expression level of mRNA was calculated with the 2^−∆∆Ct^ method, and β-actin was served as an internal control.

### Enzyme-Linked Immunosorbent Assay

Huh 7 cells were treated with UDCA in both normoxic and hypoxic conditions. After intervention, the supernatants were collected and the protein levels of VEGF and IL-8 were determined by enzyme-linked immunosorbent assay (ELISA) kits (R&D Systems, Minneapolis, MN, United States) according to the manufacturer’s protocols.

### Matrigel Plug Angiogenesis Assay *in vivo*

BALB/c nu/nu mice were administered a subcutaneous injection of 400 μL mixture containing 2 × 10^6^ EA.hy 926 cells premixed with a Corning Matrigel Matrix phenol red-free (Cat. No. 356237), IL-8 (100 ng/ml), and UDCA (50 μM). Nude mice were randomly divided into three groups (the Control group, IL-8 group, and IL-8+UDCA group). Matrigel plugs were removed and imaged after 10 days. A Bestbio reagent kit (Bestbio, CHA) was applied to detect the hemoglobin content of the matrigel plugs according to the manufacturer’s protocol. Plugs were also snap frozen in the presence of optimum cutting temperature medium before sectioning and stained by hematoxylin and eosin (HE). Immunohistochemistry (IHC) assay was also performed to determine the expression of CD31, VEGF, and vWF (Santa Cruz Biotechnology, CA). Guide for the Care and Use of Laboratory Animals of the National Institutes of Health was strictly complying with in the *in vivo* experiment which was approved by the Committee on the Ethics of Animal Experiments of the Second Military Medical University.

### Western Blot Assay

Total protein was extracted with a cell lysis buffer containing proteinase inhibitors. A BCA assay kit (Thermo, United States) was used to quantify the protein concentrations. Then, the proteins were denatured and size fractionated by 10% SDS-PAGE and transferred to PVDF membranes (Millipore, United States). After blocking, the membranes were incubated with primary antibodies at 4°C overnight. For detection, secondary antibodies conjugated to horseradish peroxidase were incubated at room temperature for 2 h, and band signals were visualized by enhanced chemiluminescence reagents (Thermo, United States).

### Dual-Luciferase Reporter Assay

Huh 7 cells were plated into 24-well plates and transiently co-transfected with the HRE-luciferase reporter plasmid and pRL-TK plasmid when the cells reached an approximately 70–80% confluence. The transfection medium was replaced with a complete DMEM medium after 6 h and incubated under normoxic or hypoxic conditions for additional 18 h. Cells were treated with or without UDCA for 24 h and then were lysed with a passive lysis buffer, and the dual-luciferase reporter assay system (Promega, Madison, United States) was applied to assess the reporter gene expression. The relative fluorescence intensity of each group was presented as the ratio of intensity of firefly fluorescence/intensity of Renilla fluorescence, as previously described (Zhao et al., 2014).

### Statistical Analysis

Data are presented as means ± SD. Statistical analyses were carried out with SPSS 19.0 software using one-way ANOVA followed by Turkey’s test. Unless otherwise stated, *p* < 0.05 was considered statistically significant.

## Results

### UDCA Inhibited HCC Cell–Induced Angiogenesis Under Hypoxic Conditions

**FIGURE 1:**
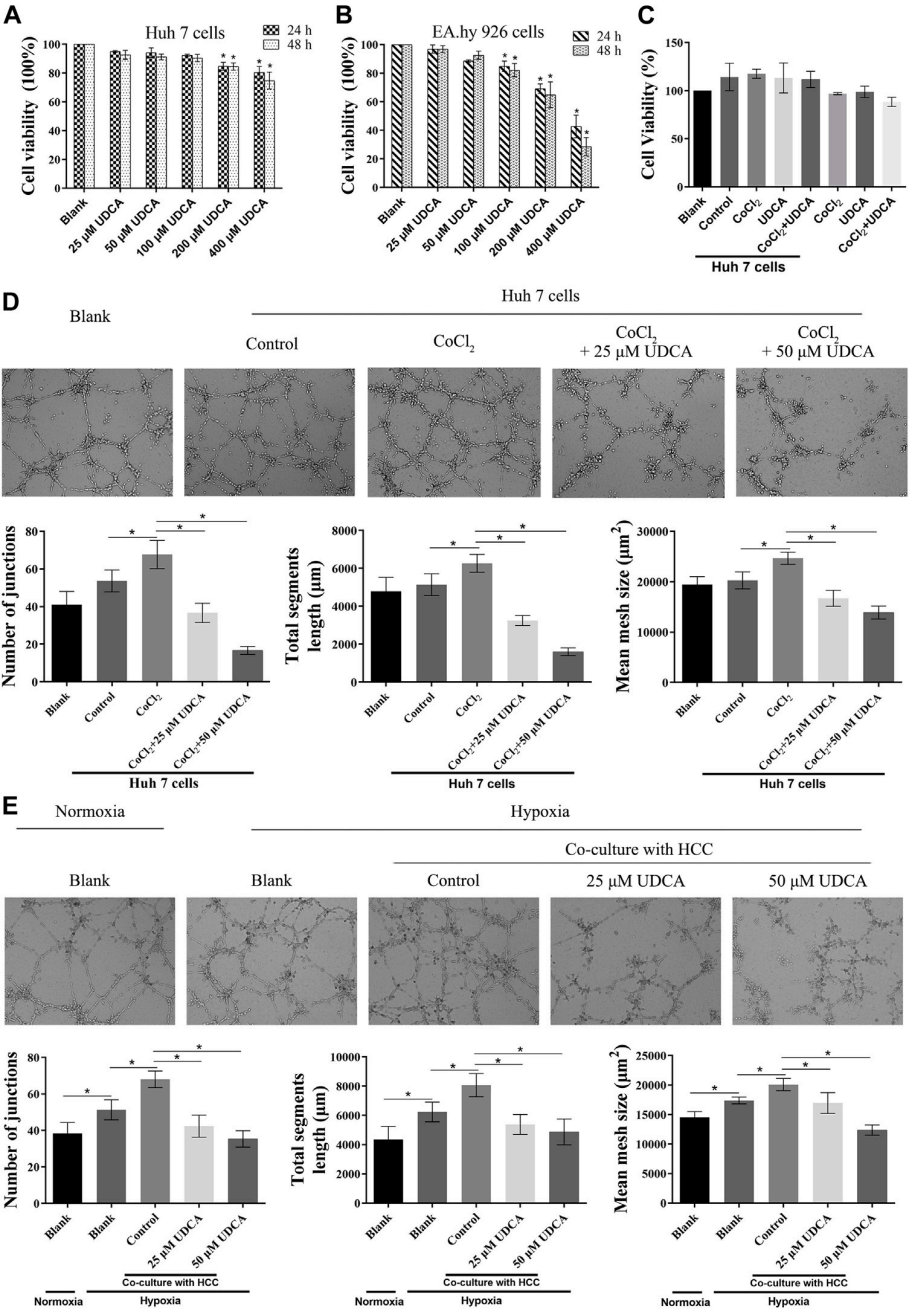
UDCA inhibited HCC cell–induced angiogenesis under hypoxic conditions. MTT assay was performed to evaluate the effect of UDCA on the proliferation of **(A)** Huh 7 cells and **(B)** EA.hy 926 cells under normoxic conditions. **(C)** Effect of UDCA on HCC cell–induced proliferation of EA.hy 926 cells under hypoxic conditions. EA.hy 926 cells were co-cultured with Huh 7 cells treated with CoCl_2_ (50 μM), UDCA (50 μM), or CoCl2 + UDCA for 24 h. And then, MTT assay was performed to evaluate the proliferation of EA.hy 926 cells. The effect of UDCA on the tube formation of EA.hy 926 cells co-cultured with Huh 7 **(D, E)** Cells treated with CoCl_2_ (50 μM) or hypoxia. The number of junctions, total segment length, and mean mesh size were calculated with ImageJ to quantify the tube formation. Data are expressed as means ± S.D, and all experiments were repeated three times. *p < 0.05 *vs.* Blank group or as indicated in the figure.

To investigate whether UDCA could inhibit the growth of the Huh 7 cells and EA.hy 926 cells, we first evaluated the cytotoxicity of UDCA on Huh 7 and EA.hy 926 cells with MTT assay. As shown in Figures 1A, B, UDCA significantly inhibited the viability of both Huh 7 and EA.hy 926 cells at high concentrations (>100 μM). Therefore, we chose 25 and 50 μM of UDCA for the following experiments. Treatment with UDCA (50 μM) for 24 h did not influence the viability of EA.hy 926 cells, with or without CoCl_2_ (50 μM) (Figure 1C). A co-culture with Huh 7 cells promoted the viability of EA.hy 926 cells. However, treatment with UDCA, CoCl_2_, or CoCl_2_+UDCA did not affect the viability of EA.hy 926 cells in the presence of Huh 7 cells.

To evaluate the effect of UDCA on HCC cell–induced angiogenesis, we performed tube formation assay under hypoxic conditions. Under normoxic conditions, co-culture with Huh 7 cells did not significantly promote the tube formation of EA.hy 926 cells (*p* > 0.05) (Figure 1D). However, co-culture with Huh 7 cells under hypoxic conditions significantly increased the tube formation of EA.hy 926 cells (*p* < 0.05) (Figures 1D,E). UDCA (25 and 50 µM) antagonized the effects of Huh 7 cells on the tube formation of EA.hy 926 cells under hypoxic conditions (Figures 1D,E).

### UDCA Inhibited Hypoxia-Induced VEGF and IL-8 Expression in HCC Cells

**FIGURE 2:**
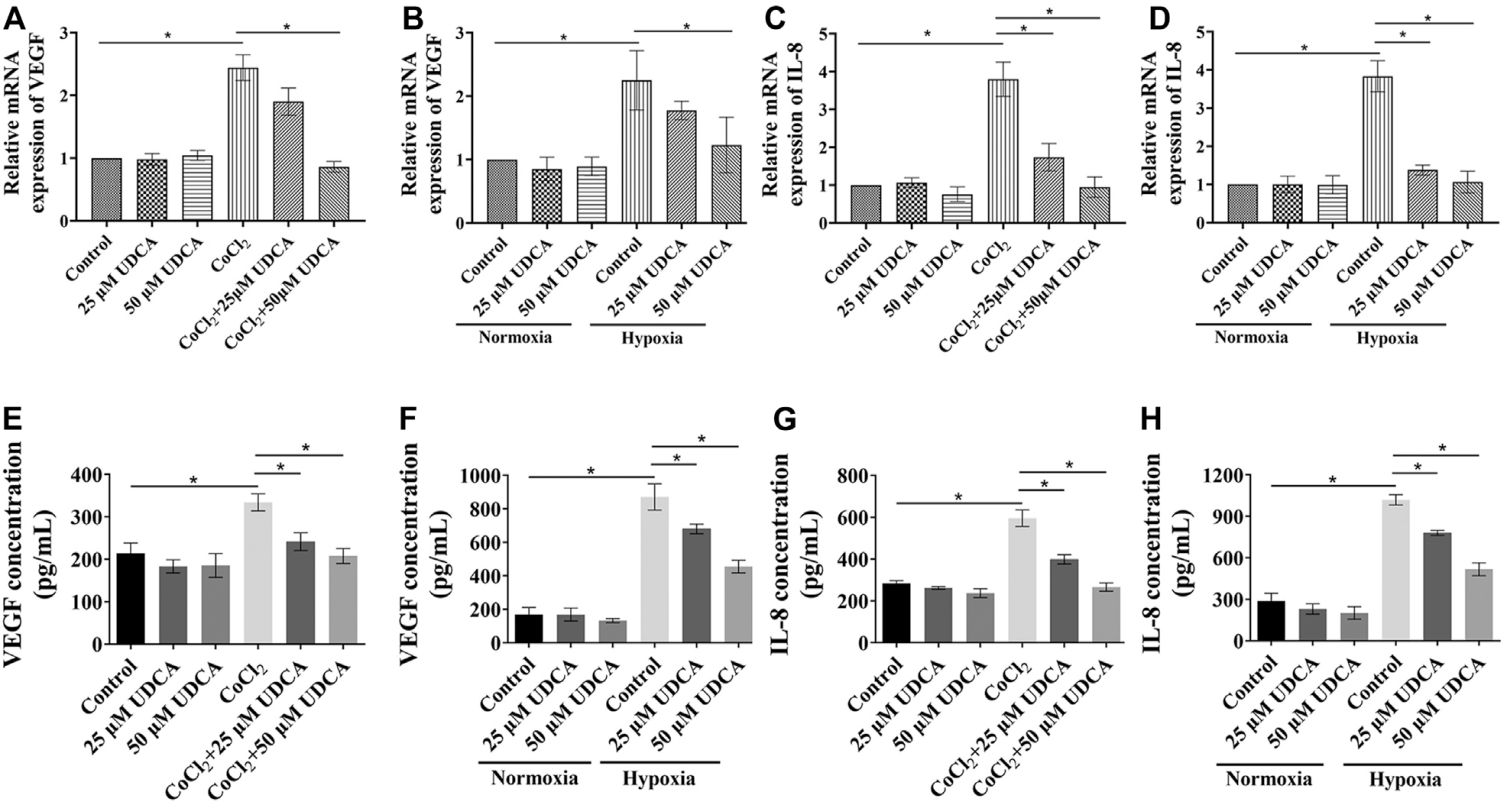
UDCA inhibited hypoxia-induced VEGF and the IL-8 expression in HCC cells. The effect of UDCA on the VEGF or IL-8 mRNA levels of Huh 7 cells with **(A)** or **(E)** CoCl_2_ or **(B)** or **(F)** hypoxia treatment for 24 h. The VEGF and IL-8 concentrations in the supernatant of Huh 7 cells with **(C)** or **(G)** CoCl_2_ or **(D)** or **(H)** hypoxia treatment was determined by ELISA. Data are expressed as means ± S.D, and all experiments were repeated three times. *p < 0.05 as indicated in the figure.

In order to explore the mechanisms of Huh 7 cells in promoting tube formation under hypoxic conditions, the expression of VEGF and IL-8 was determined by RT-PCR and ELISA. Our previous study showed bear bile powder (BBP) could inhibit hypoxia-induced IL-8 overexpression and angiogenesis. As the main active ingredient of BBP, we next investigated whether UDCA could also inhibit angiogenesis through regulating the IL-8 expression. There were no significant changes of VEGF or IL-8 mRNA in Huh 7 cells between control and UDCA groups under normoxic conditions (Figures 2A–D). Upon hypoxic conditions (CoCl_2_ or hypoxia incubator), the levels of VEGF and IL-8 mRNA and protein were significantly upregulated compared with those under normoxic conditions (Figures 2A-H). The elevation of VEGF and IL-8 mRNA and protein were partly reversed by UDCA treatment, suggesting that UDCA may inhibit hypoxic HCC-induced tube formation by suppressing the hypoxia-induced overexpression of VEGF and IL-8.

### UDCA Inhibited IL-8–Induced Angiogenesis *in vitro* and *in vivo*

**FIGURE 3:**
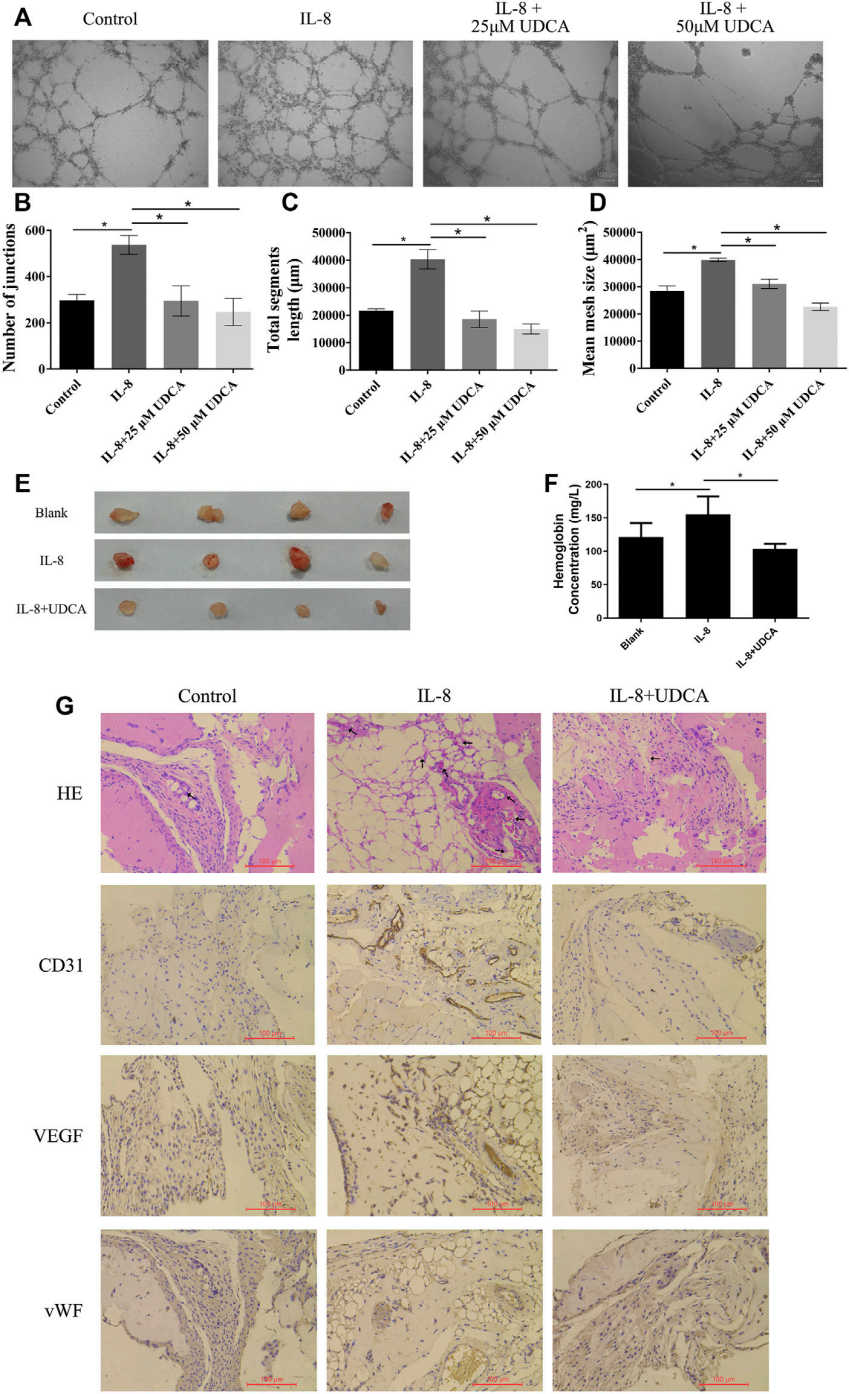
UDCA inhibited IL-8–induced angiogenesis *in vitro* and *in vivo*. **(A-D)** Effect of UDCA on IL-8–induced tube formation of EA.hy 926 cells *in vitro* and the number of junctions, total segment length, and mean mesh size were calculated with ImageJ. **(E)** Effect of UDCA on IL-8–induced angiogenesis *in vivo*. EA.hy 926 cells were treated with 100 ng/ml IL-8 and 50 μM UDCA and matrigel plug assay was conducted *in vivo*. **(F)** Hemoglobin content of the matrigel plugs determined by the Bestbio reagent kit. **(G)** Representative images of H&E staining and IHC staining of CD31, VEGF, and vWF. Data are expressed as means ± S.D. *p < 0.05 as indicated in the figure.

To further investigate the role of UDCA on IL-8–induced tube formation, we used a recombinant human IL-8 protein (R&D Systems, United States) in the next *in vitro* and *in vivo* studies. Consistent with previous reports (Feng et al., 2018), IL-8 obviously promoted the tube formation, and UDCA treatment (25 and 50 μM) significantly inhibited IL-8–induced tube formation *in vitro* (Figures 3A–D).

We next performed the matrigel plug angiogenesis assay to determine the *in vivo* anti-angiogenesis effect of UDCA. In the IL-8 group, the newly formed blood vessels were more than those in the control group and were suppressed by UDCA (Figure 3E). The hemoglobin content of the IL-8 group was higher than that of control (Figure 3F). UDCA treatment significantly decreased the hemoglobin content (*p* < 0.05). In addition, the expression of CD31, VEGF, and vWF was all upregulated in the IL-8 group and was suppressed by UDCA (Figure 3G).

**FIGURE 4:**
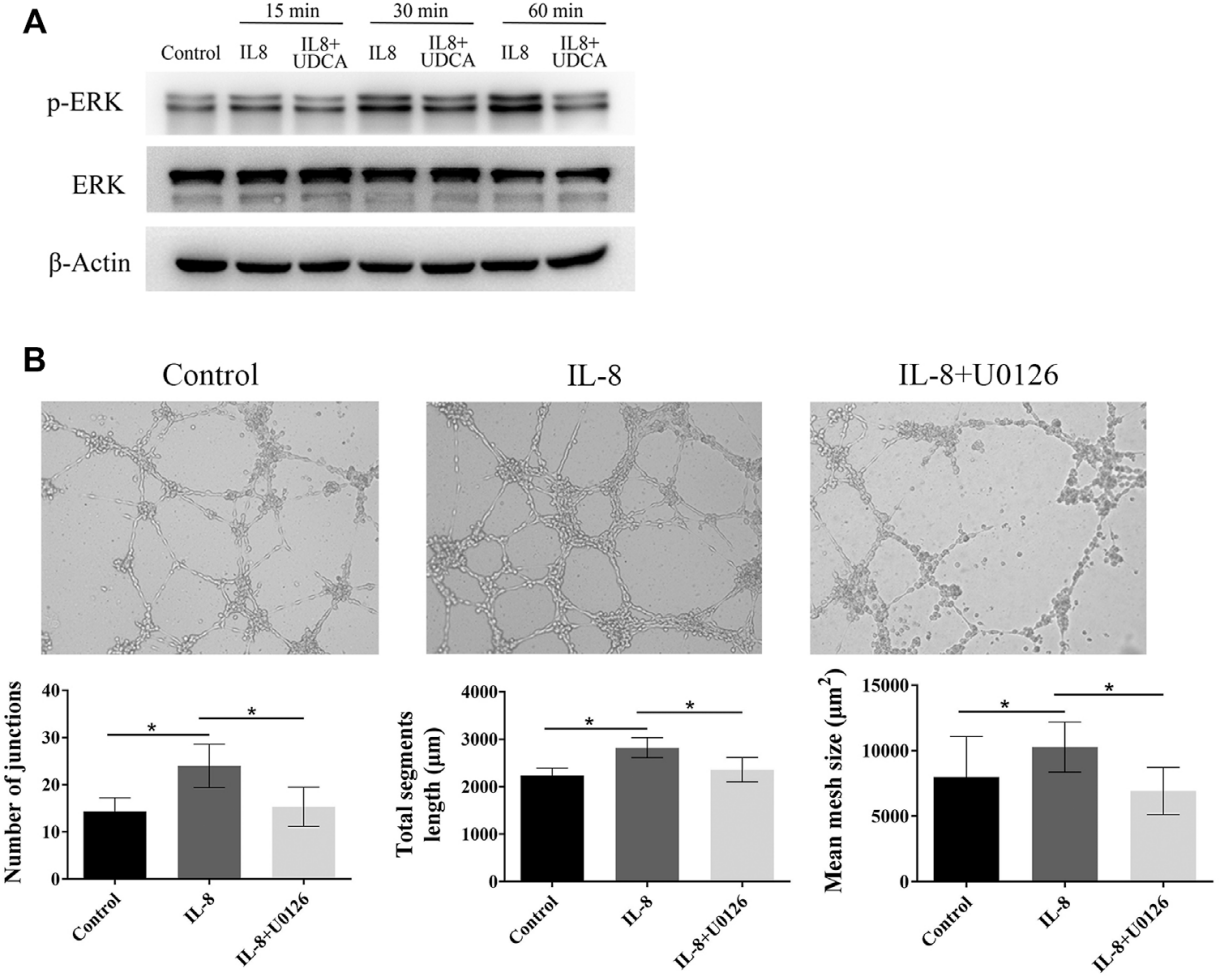
UDCA inhibited IL-8–induced angiogenesis by suppressing ERK activation. **(A)** Levels of *p*-ERK and ERK were detected in EA.hy 926 cells after treatment with IL-8 or IL-8+UDCA for indicated times. **(B)** Tube formation assay was performed to detect the effect of U0126 on IL-8–induced angiogenesis. The number of junctions, total segment length, and mean mesh size were calculated with ImageJ. Data are expressed as means ± S.D, and all experiments were repeated three times. *p < 0.05 as indicated in the figure.

In EA.hy 926 cells, IL-8 treatment induced a fast phosphorylation of ERK within 15–60 min period in a time-dependent manner (Figure 4A). UDCA obviously inhibited IL-8–induced phosphorylation of ERK. In addition, IL-8–induced tube formation *in vitro* was antagonized by U0126, indicating that UDCA may inhibit IL-8–induced angiogenesis by suppressing phosphorylation of ERK (Figure 4B).

### UDCA Inhibited HIF-1α Accumulation and Activity in HCC Cells Under Hypoxic Conditions

**FIGURE 5:**
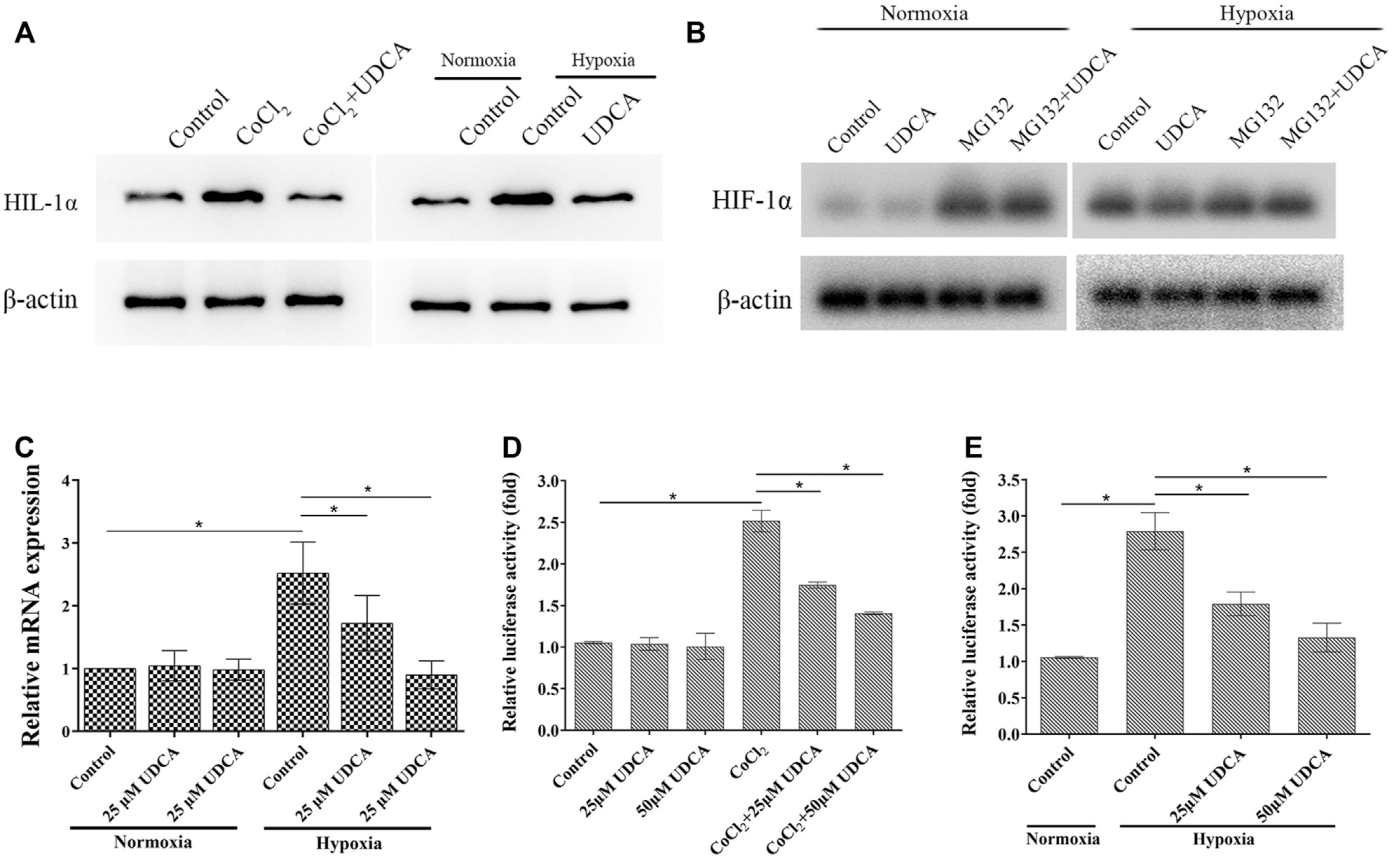
UDCA inhibited HIF-1α accumulation and activity in HCC cells under hypoxic conditions. **(A)** Effect of UDCA on the protein levels of HIF-1α in HCC cells under hypoxic conditions. Huh 7 cells were treated with UDCA under normoxic or hypoxic conditions for 24 h, and then, Western blot was performed to determine the protein of HIF-1α. **(B)** Effects of UDCA and the proteasome inhibitor MG132 on the HIF-1α expression in normal and hypoxic conditions was determined by Western blot. **(C)** Effect of UDCA on the mRNA expression of HIF-1α in HCC cells under hypoxic conditions. Huh 7 cells were treated with UDCA under normoxic or hypoxic conditions for 24 h, and then, RT-PCR was performed to determine the mRNA levels of HIF-1α. **(D, E)** Effect of UDCA on the HRE reporter gene expression in Huh 7 cells with **(D)** CoCl_2_ treatment or **(E)** hypoxia. Huh 7 cells were treated with or without UDCA for 24 h under hypoxic conditions, and then, dual-luciferase reporter assay was performed. Relative fluorescence intensity of each group was presented as the ratio of intensity of the firefly fluorescence/intensity of Renilla fluorescence. *p < 0.05 as indicated in the figure.

HIF-1α accumulates under hypoxic conditions and is associated with tumor progression. To explore the molecular target of UDCA on hypoxia-induced angiogenesis, we next determined the effect of UDCA on the HIF-1α accumulation. As indicated in Figure 5A, the HIF-1α level was upregulated under hypoxic conditions, which was inhibited by UDCA.

The level of HIF-1α is largely regulated oxygen dependently by the ubiquitin/proteasome-mediated process (Xu and Li, 2021). To further verify whether UDCA promoted HIF-1α degradation through the ubiquitin–proteasome pathway, the proteasome inhibitor MG132 was applied. Under hypoxic conditions, UDCA downregulated the level of HIF-1α, while MG132 partially antagonized the effect of UDCA on HIF-1α degradation (Figure 5B). Under hypoxic conditions, the level of HIF-1α mRNA was also significantly elevated (Figure 5C). UDCA treatment significantly downregulated the level of HIF-1α mRNA. In contrast, there were no changes of HIF-1α mRNA after UDCA treatment (25 or 50 μM) under normoxic conditions. These results indicate UDCA may not only promote the degradation of HIF-1α but also suppress the transcription of HIF-1α and thereby decrease the formation of HIF-1α protein.

After translocated to nuclear, HIF-1α binds to the hypoxia-response element (HRE) and then regulates the transcription of target genes. Therefore, we next examined the effect of UDCA on the HRE reporter gene by the dual-luciferase reporter assay in Huh 7 cells. Under normoxic conditions, the relative luciferase activity of Huh 7 cells was very low with or without UDCA treatment (Figure 5D). Both CoCl_2_ treatment and hypoxia significantly increased the HRE activities, and UDCA partially abolished these effects (Figures 5D,E).

### UDCA Inhibited the Phosphorylation of AKT and ERK in Hypoxic Conditions

**FIGURE 6:**
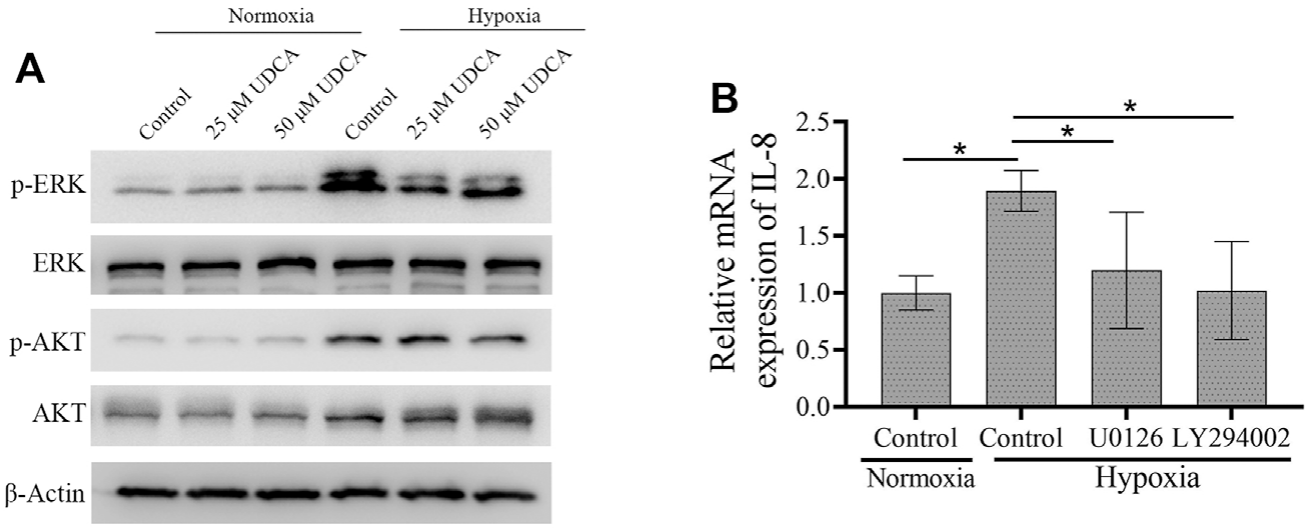
UDCA inhibited the phosphorylation of AKT and ERK in hypoxic conditions. **(A)** Western blot was applied to detect the phosphorylation of ERK and AKT in Huh 7 cells treated with or without UDCA for 24 h under hypoxic conditions. **(B)** RT-PCR assay was applied to determine the IL-8 mRNA expression with the PI3K inhibitor LY294002 or MEK inhibitor U0126 treatment.

The expression of HIF-1α is regulated by PI3K-AKT and ERK-MAPK pathways (Kanda et al., 2006; Zhang et al., 2018). To further explore the mechanisms of UDCA on HIF-1α synthesis, we then investigated the phosphorylation of AKT and ERK in normoxic and hypoxic conditions. The phosphorylation of ERK and AKT was obviously upregulated in the hypoxic condition, which was suppressed by the treatment of UDCA (Figure 6A). To explore whether the downregulation of HIF-1α mRNA by the inhibition of AKT and ERK may lead to the downstream genes, we then determined the IL-8 levels after blocking the AKT and ERK activation. RT-PCR showed that upregulation of IL-8 mRNA in hypoxic conditions was partly reversed by both the PI3K inhibitor LY294002 and MEK inhibitor U0126 (Figure 6B).

### Overexpression of HIF-1α Reversed the Effects of UDCA on Hypoxic HCC Cell–Induced Angiogenesis

**FIGURE 7:**
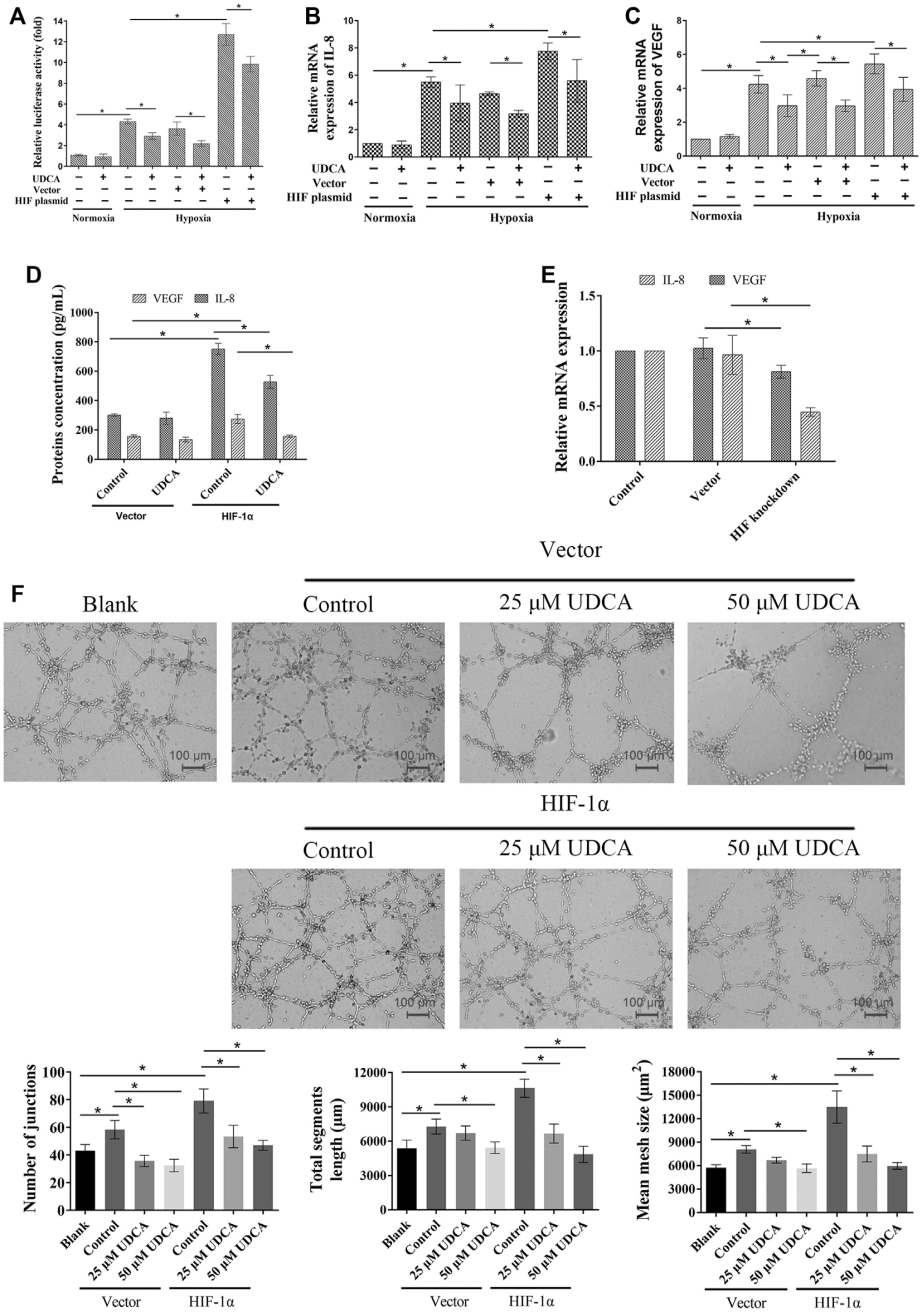
Overexpression of HIF-1α partly reversed the effects of UDCA on the expression of VEGF and IL-8– and HCC cell–induced angiogenesis. **(A)** Dual-luciferase reporter assay was performed to determine the HRE reporter gene expression in Huh 7 cells after HIF-1α overexpression plasmid transfection (Huh 7-HIF-1α). Real time RT-PCR was applied to detect the mRNA of IL-8 **(B)** and VEGF **(C)**. **(D)** ELISA assay was performed to determine the VEGF and IL-8 concentrations. **(E)** HIF-1α expression of Huh 7 cells was silenced and cultured in the hypoxia condition, and IL-8 and VEGF mRNA were detected. **(F)** HIF-1α overexpression antagonized the inhibitory effect of UDCA on Huh 7-induced tube formation. The blank group means the tube formation of EA.hy 926 cells itself, while the control group means tube formation of EA.hy 926 cells co-cultured with Huh 7 cells. The number of junctions, total segment length, and mean mesh size were calculated with ImageJ. Data are expressed as means ± S.D, and all experiments were repeated three times. *p < 0.05 as indicated in the figure.

To further verify the role of HIF-1α in the inhibitory effect of UDCA on hypoxic HCC cell–induced angiogenesis, the HIF-1α overexpression plasmid and vector were transfected into Huh 7 cells, respectively. There was an approximately 13-fold increase of HER-driven luciferase activity in Huh 7 cells transfected with the HIF-1α plasmid compared with the vector (Figure 7A). Transfection of the HIF-1α plasmid completely antagonized the inhibitory effect of UDCA on HRE activity. In addition, the HRE-driven luciferase activity was also significantly inhibited by UDCA even after HIF-1α plasmid transfection (*p* < 0.05). Furthermore, the inhibitory effects of UDCA on hypoxia-induced IL-8 and VEGF upregulation were also abolished by HIF-1α plasmid transfection (Figures 7B–D). To further demonstrate the upregulation of VEGF and IL-8 by HIF-1α, we silenced the expression of HIF-1α in hypoxic conditions, and the results indicated that both VEGF and IL-8 were downregulated by the knockdown of HIF-1α, which was shown in Figure 7E.

Additionally, the tube formation assay showed that transfection with HIF-1α also partly reversed the effect of UDCA on Huh 7 cell–induced tube formation under hypoxic conditions (Figure 7F).

## Discussion

Hepatic endothelial cell–mediated angiogenesis plays an important role that it not only provides oxygen but also helps the HCC cells escape to a new organ (Adewole, 2020; Shang et al., 2020). In a hypoxic microenvironment, HCC cells may switch to anaerobic metabolism, create a more aggressive phenotype, and influence the stroma cells. In the current study, we found that Huh 7 cells in hypoxic conditions increased the tube formation of EA.hy 926 cells through overexpressed VEGF and IL-8. UDCA, a licensed agent for primary biliary cholangitis, antagonized hypoxic HCC cell–induced angiogenesis and inhibited the upregulation of HIF-1α, VEGF, and IL-8. UDCA also suppressed the HRE-driven luciferase activity in hypoxic Huh 7 cells. All these effects of UDCA were blunted by the transfection of the HIF-1α overexpression plasmid. Our results also showed that UDCA inhibited hypoxic Huh 7 cell–induced angiogenesis by the inactivation of AKT and ERK signaling. Furthermore, UDCA also inhibited IL-8–induced angiogenesis through the ERK pathway in endothelial cells.

Numerous studies have reported that angiogenesis plays a vital role in the development of HCC (Chung et al., 2010). VEGF, a well-known angiogenesis factor, has been taken as an important therapeutic target in the treatment of HCC. Many new agents used in the clinical treatment of HCC target VEGF-mediated angiogenesis, such as bevacizumab (Demir et al., 2021). However, the anti-angiogenic therapy may exacerbate hypoxia through excessive vascular pruning. HIF-1α plays a central role in hypoxia-related signaling pathways, including angiogenesis. The levels of HIF-1α were increased in both hypoxia and CoCl_2_-treated HCC cells. HIF-1α may also trigger the angiogenesis factors, such as VEGF (Liu et al., 2020; Shi et al., 2021). In the present study, we showed that hypoxic HCC cells induced a more rapid tube formation than in the normoxic condition. Therefore, inhibition of HIF-1α accumulation in HCC cells in the hypoxic microenvironment may alleviate the expression of VEGF and angiogenesis. We showed that UDCA is able to suppress the hypoxic HCC cell–induced tube formation and decrease HIF-1α and VEGF levels, indicating it may be an effective anti-angiogenesis agent in the hypoxic condition.

Additionally, HCC cells can also sustain angiogenesis in a VEGF-independent manner (Morse et al., 2019). IL-8, a member of the CXC chemokine family, is a potent and VEGF-independent mediator of angiogenesis (Li et al., 2012; Miller et al., 2020). IL-8 could also modify the HCC microenvironment by recruiting cancer-associated neutrophils that contribute to angiogenesis (Fousek et al., 2021). Several studies have demonstrated that a higher IL-8 level is associated with worse prognosis in HCC (Deng et al., 2019; Shakiba et al., 2019). IL-8 triggered the activation of AKT and ERK in endothelial cells, which is involved in angiogenesis (Heidemann et al., 2003). Results in the present study indicate that hypoxia upregulates the expression of IL-8. Consistent with previous studies, IL-8 exerted a direct role in promoting angiogenesis. UDCA not only inhibited hypoxia-induced IL-8 upregulation but also suppressed IL-8–induced angiogenesis both *in vitro* and *in vivo*. UDCA also suppressed IL-8–induced phosphorylation of ERK in endothelial cells. These results suggest UDCA may not only inhibit hypoxia-induced VEGF upregulation but also suppress hypoxia-induced IL-8 elevation and IL-8–induced angiogenesis through ERK.

In normoxic conditions, HIF-1α is rapidly hydroxylated by prolyl hydroxylases and subsequently ubiquitylated and degraded, while HIF-1α is stabilized in the hypoxic condition. Our study showed that MG132, an inhibitor of the proteasome, partially antagonized the inhibitory effect of UDCA on HIF-1α, indicating that UDCA may promote the degradation of HIF-1α. In addition, AKT and ERK are two important molecules in the regulation of the HIF-1α expression. Activation of AKT and ERK increases the expression of HIF-1α in HCC cells and thereby, promotes the secretion of VEGF (Roychowdhury et al., 2018; Cao et al., 2019). Our study showed that UDCA may inhibit the transcription of HIF-1α and the phosphorylation of AKT and ERK of HCC cells in hypoxic conditions, which indicate that UDCA may hinder the protein synthesis of HIF-1α. Our data suggest that UDCA is not only able to promote the degradation of HIF-1α but also inhibit the synthesis of HIF-1α and thereby inhibits the HIF-1α–driven HRE-luc reporter gene expression and tube formation.

## Conclusion

In conclusion, the hypoxic HCC microenvironment causes the upregulation of HIF-1α, thereby promoting the secretion of VEGF and IL-8, and then triggers VEGF and IL-8–mediated angiogenesis. UDCA may inhibit hypoxia-induced VEGF and IL-8 secretion by HCC cells and angiogenesis by downregulating HIF-1α. UDCA not only inhibits the synthesis of HIF-1α by suppressing the phosphorylation of AKT and ERK but also promotes the degradation of HIF-1α through the proteasome pathway. In addition, UDCA could also repress IL-8–induced angiogenesis by suppressing the activation of ERK. Therefore, we concluded that UDCA may inhibit hypoxic HCC cell–induced angiogenesis through suppressing HIF-1α/VEGF/IL-8–mediated intercellular signaling between HCC cells and endothelial cells.

## Data Availability

The raw data supporting the conclusions of this article will be made available by the authors, without undue reservation.
